# Self-Determined Motivation and Competitive Anxiety in Athletes/Students: A Probabilistic Study Using Bayesian Networks

**DOI:** 10.3389/fpsyg.2019.01947

**Published:** 2019-09-06

**Authors:** Francisco Javier Ponseti, Pedro L. Almeida, Joao Lameiras, Bruno Martins, Aurelio Olmedilla, Jeanette López-Walle, Orlando Reyes, Alexandre Garcia-Mas

**Affiliations:** ^1^Department of Pedagogy, University of the Balearic Islands, Palma, Spain; ^2^Departamento de Psicologia Social e Organizacional, ISPA – Instituto Universitario, Lisbon, Portugal; ^3^Portuguese Athletics Federation, Lisbon, Portugal; ^4^GICAFE de la UIB, University of Lisbon, Lisbon, Portugal; ^5^Departamento de Personalidad, Evaluación y Tratamiento Psicológicos, University of Murcia, Murcia, Spain; ^6^Facultad de Organización Deportiva, Universidad Autónoma de Nuevo León, Nuevo León, Mexico; ^7^Department of Psychology, University of the Balearic Islands, Palma, Spain

**Keywords:** Bayesian networks, self-determined motivation, competitive anxiety, athletes, students

## Abstract

This study attempts to analyze the relationship between two key psychological variables associated with performance in sports – Self-Determined Motivation and Competitive Anxiety – through Bayesian Networks (BN) analysis. We analyzed 674 university students that are athletes from 44 universities that competed at the University Games in Mexico, with an average age of 21 years (SD = 2.07) and with a mean of 8.61 years’ (SD = 5.15) experience in sports. Methods: Regarding the data analysis, firstly, classification using the CHAID algorithm was carried out to determine the dependence links between variables; Secondly, a BN was developed to reduce the uncertainty in the relationships between the two key psychological variables. The validation of the BN revealed AUC values ranging from 0.5 to 0.92. Subsequently, various instantiations were performed with hypothetical values applied to the “bottom” variables. Results showed two probability trees that have extrinsic motivation and amotivation at the top, while the anxiety/activation due to worries about performance was at the bottom of the probabilities. The instantiations carried out support the existence of these probabilistic relationships, demonstrating their scarce influence on anxiety about competition generated by the intrinsic motivation, and the complex probabilistic effect of introjected and identified regulation regarding the appearance of anxiety due to worry about performance.

## Introduction

One of the biggest problems facing social science is the great difficulty involved in predicting human behavior. It may be due to the large number of variables that bear influence on it, however, other explanations have also been proposed within the framework of information theory (IT) and the entropy inherent in the development of open and closed systems, although they do not form part of the currently valid paradigm in psychological research (Luce, 2003; Chen and Huang, 2018; Olmedilla et al., 2018). As far as statistical methods in Psychology are concerned, something very similar to the analysis derived from Bayes’ theorem has arisen, which has a very direct relationship with the classic concept of Entropy (Laming, 2001; Farr et al., 2018). Adding sense to the similarity between these two concepts, we can argue that “IT provides a constructive criterion for setting up probability distributions on the basis of partial knowledge, and leads to a type of statistical inference that is called the (Jaakkola et al., 2000).

Therefore, it is plausible to assume that the conceptual framework of entropy is quite fitting when attempting to explain the situation of an athlete before a competition at a given moment in time. The so-called entropic “time arrow” – which implies irreversibility of events, by prohibiting the symmetry between past and future – understood psychologically, implies the generation of a past, through past events recall and memory-building, in the face of a mobile future that is configured mainly on the basis of expectations and a broad spectrum of emotions ranging from hope to fear.

In the case of sports psychology, one of the most relevant issues already extensively studied but still open to discussion affects precisely these two aspects of the “time arrow”: how do emotions associated with future performance combine with the athletes’ motivational past?

Motivation is one of the most studied variables in the context of sport (Lindahl et al., 2015; Clancy et al., 2016), and it is defined as the cause of a behavior, which operates at a psychological level within the individual and determines the execution or not of a certain activity (Tohidi and Jabbari, 2012). Intrinsic motivation can be defined as one in which individuals move autonomously toward new challenges, broader experience frameworks, and greater coherence in understanding. It represents a behavior that interests them, seeks encouragement, limits evidence, and openly assimilates novelty. On the other hand, there are four motivational norms that offer a broader framework on external motivation. The first of these is external regulation, in which the individual regulates his behavior through externally controlled rewards and punishments; the second is introjected regulation; in that regulation, the individual, by complying with internal demands, can develop certain forms of self-esteem, self-satisfaction and feelings of pride about himself; in identified regulation people value the importance of certain behavior and see it as something important for themselves; in integrated regulation, it implies that the individual brings a value or regulation in congruence with the other aspects of himself; with his basic psychological needs and with his other identifications. Finally, a motivation describes a state in which the individual is not motivated to behave or behaves in a way that is not mediated by intentionality (Ryan and Deci, 2017).

On the other hand, another of the most widely studied variables in regard to the activation of individual and sports performance is anxiety (Patel et al., 2010; Ponseti F. et al., 2016). This is defined as an immediate emotional state, characterized by apprehension and tension, associated with the activation of the organism that occurs in situations of competition (Martens, 1977). Also, competitive anxiety has been characterized by two cognitive components. One of these is worry, which is understood as restlessness about the potentially negative consequences associated with poor performance; and the second is de-concentration, which is associated with the athlete’s difficulty to focus on key aspects of the task to be performed that impede clarity of thought during the competitive situation (Grossbard et al., 2009).

That is why, in view of the scarcity of relevant literature (Fuster-Parra et al., 2015), the objective of this paper is to investigate the relationships that exist between motivational regulations (intrinsic regulation, integrated regulation, identified regulation, introjected regulation, external regulation, and amotivation) and anxiety (cognitive anxiety, worry, and de-concentration), in relation to competition in sports. The study of the relationship between these variables is of particular importance, since some authors (Nuñez and García-Mas, 2017) even affirm the possibility that anxiety in its different dimensions (e.g., cognitive, somatic, and motor) in certain conditions and based on the intrinsic characteristics of the individuals, such as their motivational regulation, can play a facilitating role of performance in competitive contexts.

In our study, framed within a closed IT system (a sports competition directed at a homogenous sample of athletes/students), we decided in favor of using a tool recommended for this type of situation, namely analysis through a machine learning classification and Bayesian networks (BN) (Puga et al., 2007; Trafimow and Marks, 2015). This approach should allow us – in part – to simulate the reversal of the “time arrow” when modifying parameters in the temporal succession of events based on the principles of probability inherent to the BNs, which presuppose independence between the events under study.

Bayesian networks are beginning to be more widely used in the field of Social Science (Ranganathan et al., 2014; Lewandowski et al., 2015; Fuster-Parra et al., 2017; Chen and Huang, 2018) and, more recently, they have been introduced as a useful methodology in Sports Psychology, given their ability to provide information on the probability of occurrence of events (some of them psychological) related to performance in sports or, for example, the likelihood of sports injuries. BN have been used to discover relationships between negative features in sport, co-operative team work, motivation and types of sporting cooperation among players on competing teams, motivational climate and anxiety (Jones and Hanton, 2001) and relative age effect (Fuster-Parra et al., 2014, 2016; Ishigami, 2016; Ponseti F.J. et al., 2016).

Before the application of the BN, a machine learning classification model, namely CHAID (Chi-squared Automatic Interaction Detector), is used to discover the dependence links between anxiety and motivation.

As indicated, the psychological variables selected (the motivational and anxiety features of competing athletes/students) are located on both sides of the fulcrum of the present, between past and future. On the basis of the rationale expressed above, our aim in this study is to ascertain the probabilistic links between the different factors of self-determined motivation (Li et al., 2017) and those related to the anxiety associated with competition in young athletes from different sports specialties, especially to attempt to reduce the likelihood of anxiety occurring, and then to interpret the results obtained according to the entropy inherent in the system under study.

## Materials and Methods

### Participants

The study was performed in Mexico with 674 university students that are athletes who competed in the University Games (Universiade) in 2017. They were from 44 universities, at least one athlete from each participating university, with a mean age of 21 years old (SD = 2.07) and a mean sports experience of 8.61 years (SD = 5.15). All universities took part in this study. All participants were previously informed about the protocol and purposes of the study. The study protocol was approved by the local ethics committee of the Universidad Autónoma de Nuevo León (Mexico) in accordance with current ethical standards in sport and scientific research.

### Instruments

Competitive anxiety in sport was measured using the Spanish language adaptation (*Escala de Ansiedad Competitiva*), (Pineda-Espejel et al., 2016) of the Sport Anxiety Scale 2 (SAS-2) (Smith et al., 2006). The SAS-2 consists of three 5-item scales to measure three factors: somatic anxiety, worry, and lack of concentration or de-concentration. Each item was answered on a 4-point Likert scale with a range between “not at all” and “very much.”

Self-determined motivation. An adapted version of the Sports Motivation Scale (Pelletier et al., 2013) was used in this study. This measure had been previously translated into Spanish cpssnm(Martín-Albo et al., 2006). The SMS-II is an 18-item inventory comprising six factors of behavioral regulation. These factors were extracted from the Self-determination Theory to test a model that would enable us to assess Autonomous and Controlled Motivation. The subscales are intrinsic motivation (e.g., “*for the pleasure it gives me to know more about the sport I play*”); identified regulation (e.g., “*because in my opinion it is one of the best ways to meet people*”); introjected regulation (e.g., “*because it is absolutely necessary to do sports if one wants to be in shape*”); external regulation (e.g., “*because it makes me better regarded by people I know*”); and a-motivation (e.g., “*I used to have good reasons for doing sports, but now I am wondering whether to carry on doing it*”). Each item was answered on a 7-point Likert scale with a range between “not at all” and “very much.”

### Procedure

The data was collected during the University Games (Universiade). Personal contact was first made with team coaches to inform them about the project. Instruments were applied in the concentration hotels, prior to their participation in the sporting event, at least one researcher was present to give instructions and answer athletes’ questions. Emphasis was placed on the confidentiality of athletes’ individual responses as well as on the need to answer honestly. In addition, the researcher explained to them that responding to the questionnaires implied voluntarily accepting to participate in the research.

### Data Analysis

The CHAID (Chi-squared Automatic Interaction Detector) algorithm is used to discover relationships between a categorical or ordinal dependent variable and other categorical predictors. It computes a decision tree, which includes meaningful nodes that classify a nominal or ordinal dependent variable (Magidson and Vermunt, 2005). It is a convenient way to summarize data since it makes it easy to view relationships. It relies on the Chi-square test to determine the best next split at each node of the tree. To obtain the decision tree, the R package “CHAID” was used in the dataset. To apply the algorithm, a dependent variable and the independent variables need to be chosen beforehand. We selected “somatic anxiety” as the dependent variable and the remaining variables as the independent variables.

To obtain a BN (Bayesian network), it was necessary to determine a structure (defined by a twofold method). First, through a Directed Acyclic Graph (DAG) and the conditional probabilities assigned to each node of the DAG, and secondly, restricting pathways by using the CHAID algorithm on the resulting graph. Therefore, learning a BN implies the following two tasks: (i) structural learning, i.e., the identification of the topology of the BN, and (ii), parametric learning, i.e., the estimation of numerical parameters (conditional probabilities) given a network topology.

Structural learning was used to obtain the BN, the bnlearn package (Scutari, 2010) of the R language (Rosselet, 1987). To obtain the structure, we could follow either a search and score algorithm (Korb and Nicholson, 2010), which assigns a score to each BN structure and selects the model structure with the highest score, or a constraint-based search algorithm (Spirtes et al., 1993), which establishes conditional independence analysis on the data where an undirected graph is generated and converted into a BN using an additional independence test. We used the score-based algorithm Tabu (Korb and Nicholson, 2010), which gave us a plausible model for our data. The search procedure finds the structure that best improves the score, i.e., using the highest score (Bayesian Information Criterion – BIC).

## Results

In regard to the values found in the variables under study, the lack of importance of external regulation compared with the values of intrinsic motivation, and, to a lesser extent, with those of amotivation, are relevant. The subscales of the intrinsic and internalized regulation values are high, with regulation being identified as the one with the lowest values. Descriptive data of the variables studied are shown in Table 1.

**TABLE 1:** Descriptive data of the variables studied, mean and standard deviation (*N* = 674).

**Variables**	***M***	***SD***
Somatic anxiety	1.71	0.62
Worry anxiety	2.75	0.80
Lack of concentration anxiety	1.73	0.63
Intrinsic regulation	6.22	0.18
Integrated regulation	6.13	0.56
Identified regulation	4.17	0.74
Introjected regulation	5.83	0.15
External regulation	1.61	0.65
Amotivation	2.59	0.98

When we observe the values of anxiety related to competition, we can see that they are below the average in the ranges. Anxiety related to competition shows values below the mean scores in the possible ranges in all cases. Somatic anxiety and lack of concentration are the dimensions with the lowest values, while worry about performance is higher than the average. SD values – with the exception of one case – are consistent with a relatively homogeneous sample and none of the values found is especially significant. The SD value of amotivation is very close to the mean values, indicating that answers about this variable were not overly homogeneous.

When the values for anxiety related to competition are observed, one can see that they are below the average in each range. Anxiety related to competition returns values in all cases below the mean score in the possible ranges. Somatic anxiety and lack of concentration are the dimensions with the lowest values, while concern about performance is above average. The SD in all cases are consistent with a relatively homogeneous population, and none of the values found is especially remarkable.

**FIGURE 1:**
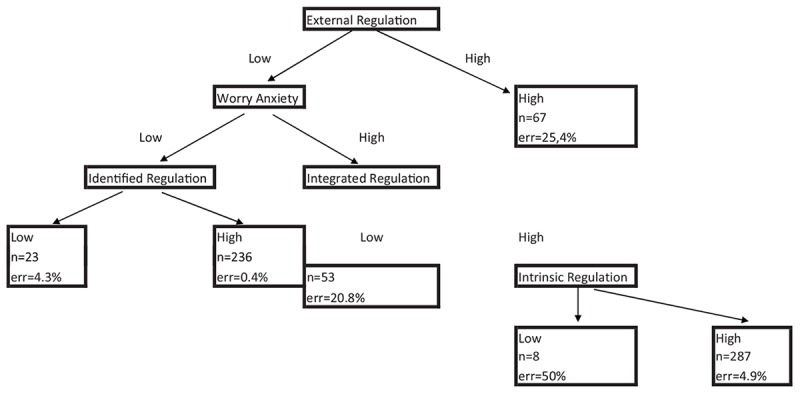
Chi-squared Automatic Interaction Detector algorithm defining the inter-dependent variables.

Figure 1 shows the result of applying the CHAID algorithm, which revealed a tree prediction model for the “Somatic Anxiety” variable; therefore, uncertainty in the data obtained was reduced, allowing for BN analysis with restrictions and thus reducing the complexity of the entire system. Five variables were found to predict “Somatic Anxiety.” Four of these were motivational: intrinsic and external global regulations, and two subscales of intrinsic motivation, identified and integrated regulation; and the last one being anxiety related to performance.

The CHAID tree starts with the top “External Regulation” decision node, with the 674 instances of the data set divided into two partitions, based on the result of splitting this node into two categories – “High” (*n* = 67) and “Low” (*n* = 607). The “Low” category shows the majority of cases are associated with “Somatic Anxiety.” This node is further split on the basis of the value of the “Anxiety from Worry about performance” predictor variable, resulting into two more nodes based on this category. Splitting continues until either the new split does not help to improve prediction accuracy, or a node contains less instances than a pre-defined size.

As for the BN, validation was performed using a 10-fold cross validation, taking into consideration the area under the curve (AUC), which is defined as the probability of correctly identifying a pair of cases (positive and negative). As can be seen in Table 2, the validation values generated in the BN with all the variables under study are acceptable. However, in the same line as the descriptive values, the minor AUC values correspond to amotivation, the identified regulation, as well as to anxiety from worry about performance. To achieve a better understanding of the accuracy of the classification, sensitivity and specificity were calculated. While it is possible to find high accuracy in some variables, when sensitivity and specificity are observed, we detect a null positive classification ability on identified regulation, low classification on intrinsic and integrated regulation, and a generally high negative classification ability.

**TABLE 2:** Validation of the BN developed with the variables studied: AUC values, accuracy, sensitivity, and specificity.

**Variables**	**AUC**	**Accuracy**	**Sensitivity**	**Specificity**
Somatic anxiety	0.79	0.93	0.97	0.62
Worry anxiety	0.59	0.64	0.39	0.80
Lack of concentration anxiety	0.83	0.94	0.98	0.68
Intrinsic regulation	0.60	0.94	0.25	0.98
Integrated regulation	0.61	0.86	0.24	0.98
Identified regulation	0.51	0.90	0.00	1
Introjected regulation	0.63	0.80	0.31	0.96
External regulation	0.84	0.95	0.98	0.70
Amotivation	0.54	0.78	0.96	0.11

**FIGURE 2:**
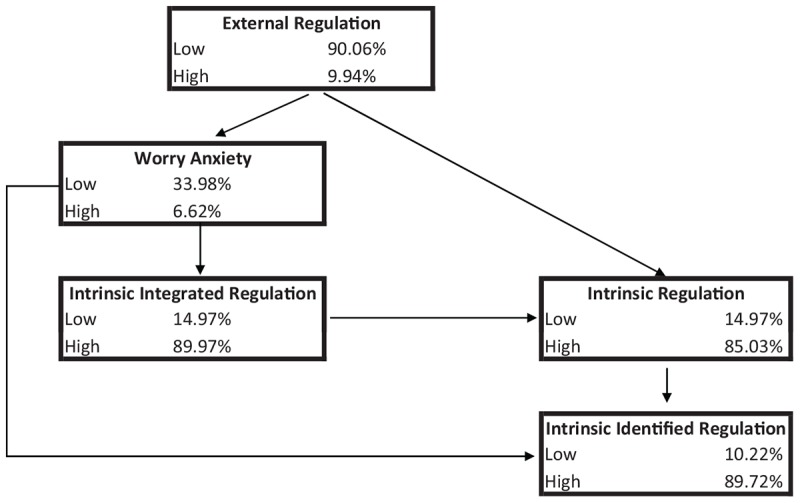
Bayesian Networks generated using the restrictions made after the CHAID algorithm (inter-dependent variables only).

Figure 2 shows the BN generated with the restrictions found after the CHAID analysis using an acyclic graph. The top variable is the external regulation, showing low probability, while the bottom variables (probabilistically dependent on the others) are the identified regulation, which in turn is dependent on global intrinsic regulation. The two intermediate variables – no nodes were found – are Anxiety from Worry about performance and the sub-variable of Identified Intrinsic regulation. The probability values found in the sample group indicate that the participants in our study have a high probability of being intrinsically motivated, with a low probability of perceiving external rewards or benefits (although this variable is shown as key to triggering the likelihood of the other variables occurring), as well as the presence of mean probability of anxiety from worry about performance, which acts as a buffer for the other variables.

### BN Instantiations With Hypothetical Data

The global Markov property was applied to maximize and minimize the likelihood values for different variables. The global Markov property states that any node X is conditionally independent of any other node given its Markov blanket (a Markov blanket of X in a BN is the set of nodes consisting of X’s parents, X’s children and other parents of X’s children). The features instantiated were in the Markov blanket of different features, therefore given its Markov blanket, each feature is independent of the remaining features. The two variables selected are: (1) the most relevant Anxiety subscale found, the one relating to worry about performance, as appears in the BNs; and (2) overall intrinsic motivation, which is the most significant node in the two BNs.

We began by attempting to maximize the likelihood of anxiety due to worry about performance occurring, in this case, on the CHAID restricted BN (see Table 3). The first two steps are the ones that produce the most significant changes; indeed, probability of “lack of concentration” and “introjected regulation” decrease to give an 18% increase in low level probability of anxiety from worry, which comes from a low value: one third of detected likelihood. The following steps have less weight and, in order of significance, include a reduction in somatic anxiety and different sub-scales for self-determined success motivation, although the latter represents a reduction of less than one tenth of the likelihood, which reaches a maximum of 64.83% (barely double the probability actually obtained in the sample, which is 35.88%). This indicates that the existence of anxiety from worry about performance is very strong and cannot be reduced even if it is forced by using hypothetical “anti-entropic” values and will only return values within the average likelihood of occurrence.

**TABLE 3:** Step-by-step instantiations leading to maximization of the likelihood of low perfomance’ Worry anxiety in the BN with CHAID restrictions.

**Step**	**Instantiaded variable**	**Level**	**Value**
1	None (initial value)	Low	35.88
2	Lack of concentration	Low	43.44
3	Introjected regulation	Low	51.00
4	Somatic anxiety	Low	54.05
5	Identified regulation	Low	57.55
6	Integrated regulation	Low	64.07
7	External regulation	Low	64.42
8	Amotivation	Low	64.83

In Table 4, it can be seen that when trying to reach the maximum probability of the identified intrinsic motivation sub-scale (which is the most relevant bottom motivational variable in the NB with restrictions), the reversed BN only achieves an increase of 6%. In this sense, it is unquestionably a very solid variable. This maximum value is reached in three steps, with very similar values, two of them imply reaching the maximum probability of overall intrinsic motivation and introjected regulation, and then to the minimum value for anxiety from worry about performance.

**TABLE 4:** Step-by-step instantiations leading to maximization of the likelihood of high identified intrinsic regulation in the BN with CHAID restrictions.

**Step**	**Instantiaded variable**	**Level**	**Value**
1	None (initial value)	high	89.16
2	Global intrinsic motivation	high	91.07
3	Perfomance worry anxiety	low	92.99
4	Introjected motivation	high	95.19

## Discussion

Our aim in this study was to ascertain the probabilistic links between the different factors of self-determined motivation and those related to anxiety, particularly attempting to reduce the likelihood of anxiety occurring. Firstly, in response to the objective and question posed, it should be noted that all the analyses carried out demonstrate that it is possible to reduce uncertainty in the relationships between motivation and anxiety in the field of competitive sports.

The main findings can be summarized by the statement that competitive anxiety is completely “disassembled” in its three factors with respect to its probabilistic weight of occurrence: the predecessor variable of the others is worry about performance, while the other two dimensions have occupied some key positions as well. De-concentration (or lack of concentration) anxiety (the one most responsible for diminished performance) acts as a “modulator” on anxiety from worry triggered by the weight of the probability of external motivation, while somatic anxiety becomes the “final” sub-product of the other variables. Further studies on our group have demonstrated the caution with which somatic signs of anxiety must be taken by external observers when determining the ability of subjects to perform their tasks (García-Mas et al., 2016).

All the analyses carried out demonstrate that it is possible to reduce uncertainty in the relationships between motivation and anxiety when in the performance of competitive sport. Participants in the sample have a high probability of being intrinsically motivated, with a low probability of perceiving external rewards or benefits.

However, it should be noted that this sample – composed of young student/athletes of medium rather than top performance level – presents several clear biases: low probability of anxiety and external regulation and amotivation, contrasting with high probability of emergence of self-determined motivation, without any clear predominance of the source of intrinsic regulation (Ntoumanis, 2001; Standage et al., 2003). As indicated in other studies (Wolf et al., 2015), competition-related anxiety cannot be taken as a whole but, rather, its three factors must be considered separately in both the psychological evaluation and intervention, as is clearly shown in the BN without restrictions for somatic anxiety.

The results found in the BN with restrictions showed that there were five variables to predict somatic anxiety. Four of them are motivational: intrinsic and external global regulations, and two sub-scales of intrinsic motivation, identified and integrated regulation, with the last one being anxiety related to performance.

The probability values found in our study group indicate that the participants in the sample have a high probability of being intrinsically motivated, with a low probability of perceiving external rewards or benefits, although this variable triggers the probability of the other variables occurring, as well as the presence of average likelihood of anxiety related to performance, which acts as a buffer for the other variables.

Working with instantiations leaves the probability landscape much clearer. To obtain the lowest possible probability value for Anxiety from Worry (which is the one least associated with decreased performance (Pulido et al., 2018), several variables need to change their likelihood. The two variables whose probable occurrence is most likely to be reduced are de-concentration and introjected regulation [which is usually accompanied by “negative” emotions, such as guilt (Fenton et al., 2016)]. To a lesser extent, the likelihood of occurrence should also be reduced for somatic signs of anxiety and for high levels of motivation, both intrinsic and extrinsic. If we combine this data with the “actual” probabilities found in the BNs and the nodal or predecessor position that anxiety from worry about performance holds, we can see that perhaps the latter is indeed the key element in the system under study. Further support for this idea is given by the fact that zero probability (100% Low) cannot be achieved, which may perhaps be indicative of a specific characteristic in this type of athlete, namely athletes that are also concerned about their academic careers (Aquilina, 2013; Vilanova and Puig, 2016) and who may have some kind of “immunity” toward external regulation, which in turn triggers a lower likelihood of somatic anxiety.

Further to the previous argument, by obtaining the maximum intrinsic regulation [the one identified as the bottom variable in both BN and which is based on a high probability value (almost 90%)], it is possible to increase its likelihood when introjected and global intrinsic regulations reach 100% probability of occurrence, and when anxiety due to worry about performance is at 100% low probability, i.e., zero. Therefore, strong opposition clearly exists between the emotion of guilt and anxiety due to worry about performance in athletes’ minds.

In an attempt to summarize the results obtained more succinctly, we can conclude that the athletes in our survey start with high values of self-motivation and low anxiety associated with competition; that the BNs performed show a probabilistic “constellation” that situates anxiety due to worry about performance and external regulation as the basic predecessors, and intrinsic regulation and somatic anxiety as the bottom variables, while amotivation and the de-concentration lack any informative value. All these findings are confirmed when hypothetical values are inserted in these key variables and we are able to see how the likelihood of other variables need to be changed in order to reach their maximum or minimum probability of occurrence. Also, the obtained results reinforce that the adapted version of the Sport Anxiety Scale (SAS-2) is an adequate and valid measure for the assessment of anxiety in young athletes.

The reduction of uncertainty derives from the successive application of two statistical models. First, the CHAID algorithm has made clear the relationships (or not) of the variables studied, simplifying the subsequent probabilistic analysis. Once the BN has been carried out, the uncertainty has been further reduced by making clear the “chains” of probabilistic impact of each variable with respect to the other relevant ones. In addition, in the same sense, the instantiations have further clarified the landscape (reinforcing some of the results and weakening some others), so that the ambiguity in the probability of occurrence of the variables has been reduced in a very relevant way.

From a practical point of view, thanks to this study we know more about how to cope with Dual Career students/athletes, regarding their motivation (mostly intrinsic, which affects the type of reinforcements and incentives to use with them); or to the relevant importance of the Worry anxiety in contrast with the lower importance of the somatic anxiety in terms of detrimental performance, which is paradoxically often the one that receives the most attention from sports professionals, including psychologists.

This study entails certain limitations, the most important being those derived from the characteristics of the sample studied, as well as the impossibility of relating the variables under study to the performance (either subjectively and/or objectively evaluated) of the athletes/students. Also, the bias that these athletes reveal in regard to their type of motivation and competitive anxiety have limited the results obtained when BNs are used with limited hypothetical values.

When considering the study as a whole, the next step in the research should be to structure the system under study in a more useful manner, firstly, to analyze the concept of “dual career” completely “from the inside,” which would be useful to understanding our athletes’ specific motivational features; and secondly, to improve the quality of the instantiations made through longitudinal and “survival” studies in order to confirm the predictions derived from this use of probabilistic BNs.

## Data Availability

All datasets generated for this study are included in the manuscript and/or the supplementary files.

## Ethics Statement

The studies involving human participants were reviewed and approved by the local ethics committee of the Universidad Autónoma de Nuevo León (Mexico). The patients/participants provided their written informed consent to participate in this study.

## Author Contributions

FP, PA, AO, and AG-M: conceptualization of the study. BM and AG-M: methodology, software, validation, and formal analysis. OR and JL-W: investigation. JL-W, AO, and BM: resources. FP: writing – original draft preparation and visualization. FP, JL, PA, and AG-M: writing – review and editing. AG-M, JL-W, and AO: supervision. All the authors contributed to the revision of the manuscript, and read and approved the presented version.

## Conflict of Interest Statement

The authors declare that the research was conducted in the absence of any commercial or financial relationships that could be construed as a potential conflict of interest.
